# The Impact of Psychological Interventions on Functioning in the Context of Borderline Personality Disorder Features for Adolescents and Young Adults: A Systematic Review and Meta‐Analysis

**DOI:** 10.1111/eip.70112

**Published:** 2026-01-06

**Authors:** Benjamin D. Brandrett, Ruchika Gajwani

**Affiliations:** ^1^ School of Health and Well Being, University of Glasgow Glasgow UK

**Keywords:** adolescents, borderline personality disorder, functioning, psychological interventions, young adults

## Abstract

**Objective:**

Adolescents recruited from clinical samples with borderline personality disorder (BPD) often experience significant functional impairment across multiple domains. Evidence indicates that borderline personality features emerging before adulthood can predict long‐term difficulties and may worsen over time. However, the role of assessment methods and the impact of psychological interventions on functional outcomes remain unclear.

**Methods:**

A systematic review and meta‐analysis of randomised controlled trials (RCTs) was conducted to evaluate the impact of psychological interventions on functioning in adolescents and young adults with BPD features. Four databases (PsycINFO, Medline, Embase and CINAHL) were searched up to June 2023.

**Results:**

From 1859 identified studies, seven trials (*N* = 657) met the inclusion criteria. Across studies, psychological interventions were associated with improvements in functioning from baseline to both post‐treatment and final follow‐up. However, when comparing specialised psychological interventions to generalist treatment as usual (TAU), differences were not statistically significant. Effect sizes were small at post‐treatment (SMD = 0.13, 95% CI = [−0.05, 0.31]) and remained small at final follow‐up (SMD = 0.12, 95% CI = [−0.08, 0.33]). Substantial heterogeneity was observed across studies, and risk of bias was noted in several trials, with only two studies rated as low risk.

**Conclusions:**

The findings suggest that both specialised psychological interventions and generalist interventions yield similar outcomes in terms of functional improvement. These results have implications for clinical service design and underscore the importance of addressing the needs of this underrepresented population. More high‐quality, large‐scale trials are needed to strengthen the evidence base.

## 1 Introduction

Borderline personality disorder (BPD) is a complex mental health condition marked by instability in interpersonal relationships, self‐image, affect and impulsivity (American Psychiatric Association 2022) and is often associated with elevated suicide rates, severe functional impairment, extensive treatment utilisation and significant societal costs (Leichsenring et al. 2011).

Over the past two decades, mounting evidence has established BPD in adolescence as both a valid and reliable diagnosis, clearly distinguishable from typical adolescent development (Chanen, Betts, et al. 2022; Hutsebaut et al. 2023). Empirical research demonstrates that adolescent and adult BPD share similar characteristics, including high comorbidity rates and comparable aetiological profiles encompassing genetic factors, maladaptive attachment patterns and exposure to trauma (Winsper 2018; Bozatello et al. 2021).

In adolescents, research suggests that BPD has an estimated prevalence of between 1% and 3% in the community, increasing to 11%–22% in outpatients and 33%–49% in inpatients (Chanen et al. 2017; Guilé et al. 2018). However, there has been a reluctance to diagnose BPD in young people. Griffiths (2011) reported that in a sample of psychiatrists in the United Kingdom, the majority felt that adolescent BPD diagnosis was inappropriate, invalid or harmful. This reluctance to recognise BPD in adolescence can lead to prolonged distress, iatrogenic complications and negative encounters with healthcare services (Bateman and Fonagy 2015; Laurenssen et al. 2013).

Similar to adult populations, adolescents with BPD features commonly experience significant functional impairment (Chanen et al. 2008). Long‐term follow‐up studies have consistently shown that adolescent BPD is associated with diminished life satisfaction, limited social support and challenges across multiple functional domains, including relationships, academic performance and occupational attainment (Winograd et al. 2008). Functional impairment has been observed across a broad range of symptomatic presentations in adolescent BPD, and evidence has shown that even the presence of one BPD feature can impact functional outcomes (Kaess et al. 2017; Thompson et al. 2018), with functioning deteriorating as adolescents transition into adulthood if left untreated (Wertz et al. 2020).

Frías et al. (2017) compared younger and older participants with BPD and observed that functional deficits were more severe in the older group. It was proposed that increased severity results from the cumulative impact of challenging life events, which may lead to avoidance of new vocational and relational opportunities. More recently, Jørgensen et al. (2024) conducted a 5‐year follow‐up of adolescents previously diagnosed with BPD and reported that general functioning remained significantly impaired, highlighting the persistence of difficulties from adolescence into early adulthood. These results support Hutsebaut et al.'s (2020) proposal that recovery in social and vocational domains should be prioritised as treatment outcomes, as progress in these areas is more strongly associated with treatment success than the resolution of BPD symptoms alone. Adopting this holistic approach addresses concerns raised by service users that psychotherapies for BPD disproportionately focus on self‐harm symptoms rather than broader functional recovery (Katsakou et al. 2012).

Extensive epidemiological data highlight that while symptomatic improvement is a component of BPD management, functional impairment often endures over time. This emphasises the significance of considering a more comprehensive approach to recovery, one that extends beyond symptomatic remission (Gunderson et al. 2011; Zanarini et al. 2012). An increasing number of randomised controlled trials (RCTs) have evaluated the impact of psychological therapies on BPD features (Jørgensen, Storebø, Bo, et al. 2021). However, functional recovery is seldom described and insufficiently prioritised in assessment, treatment and research (Ng et al. 2016; Skodol 2018). There is now widespread interest in addressing the functional challenges inherent to adolescents and young adults that experience BPD features at subthreshold and threshold levels (Chanen, Nicol, Betts, et al. 2020). Improving functioning should be a critical treatment target, particularly in adolescence and young adulthood, as Zanarini et al. (2018) found that 'excellent recovery' for BPD later in life was predicted by good vocational engagement, amongst other variables such as social network size, suggesting key treatment targets for this group.

Previous reviews by Wong et al. (2020) and Jørgensen, Storebø, Stoffers‐Winterling, et al. (2021) analyzed the impact of psychological therapies on BPD symptoms in children and adolescents. Although both meta‐analyses reviewed functioning, further descriptive evaluations of functional outcomes and an expanded inclusion of young adults would better reveal how targeted interventions affect various functional outcomes within a broader developmental period of early intervention and prevention. This is particularly important as BPD typically emerges and has its peak incidence between puberty and early adulthood (Chanen, Betts, et al. 2022), and is associated with substantial long‐term social, health, and economic burden (Fok et al. 2012; Hastrup et al. 2019). Yet, this population often experiences stigmatising attitudes and exclusion from services, which can further perpetuate iatrogenic harm and health inequalities (Ring and Lawn 2025; Moran et al. 2016). Given the long‐term impact of BPD on social, health and economic outcomes, a comprehensive review is needed. This paper will synthesise existing evidence on interventions targeting functional outcomes in adolescents and young adults, addressing a critical gap in research and treatment.

### 1.1 Aims of the Study

This systematic review aims to comprehensively analyse the existing studies that investigate functional outcomes resulting from psychological intervention in adolescents and young adults (up until age 25) displaying BPD features. Specifically, this review aims to:

1. Systematically review and synthesise existing studies that investigate the impact of psychological interventions on overall functioning in the target population.
2. Identify what psychological interventions are used and examine how functioning is evaluated.
3. Analyse the effect of psychological intervention on functional domains when compared with treatment as usual (TAU) through a meta‐analysis.
4. Evaluate the methodological quality of the studies included that examine the impact of psychological intervention on functioning within this population.

## 2 Method

### 2.1 Protocol and Registration

This review was prospectively registered (https://www.crd.york.ac.uk/prospero/display_record.php?ID=CRD42023430703) with the International Prospective Register of Systematic Reviews (PROSPERO) in accordance with Preferred Reporting Items for Systematic Reviews and Meta‐Analyses (PRISMA) statement guidelines (Page et al. 2021).

### 2.2 Search Strategy

A systematic search of published studies examining the impact of psychological therapies on functioning in adolescents with BPD features was performed on 30 June 2023 using the following databases: PsycINFO (Ovid) Medline (Ovid), Embase (Ovid), CINAHL (EBSCO). The following search strings were used: Borderline personality OR Borderline state OR Borderline personality disorder OR BPD OR Emotionally unstable personality OR EUPD OR Cluster B OR Personality disorder* AND Psycholog* treatment OR Psycholog* intervention OR Psycholog* therapy OR Psychotherap* OR Schema therapy OR Dialectical therapy OR Cognitive therapy OR Brief relational therapy OR Client‐centred therapy OR Narrative therapy OR Emotion‐focused therapy OR Psychoanalytic therapy OR Family therapy OR Gestalt therapy OR Rational‐emotive therapy OR Mentalization‐based therapy OR DBT OR Dialectical Behav* Therapy OR Mindfulness OR Early intervention OR CBT OR Eye movement desensitisation OR Guided imagery OR Psychosocial intervention OR Crisis intervention OR Psychoanalysis OR Mentalisation‐based treatment OR Telepsychotherapy OR Relaxation training OR Individual psychotherap* OR Interpersonal psychotherap* OR Psychodynamic psychotherap* OR Adolescent psychotherap* OR Experiential psychotherap* OR Short term psychotherap* OR Brief psychotherap* OR Expressive psychotherap* OR Person‐centred psychotherap* AND Adoles* OR Emerg* adult* OR Young adult* OR Young person* OR Young people OR Teen* OR Youth OR Juvenile OR Child*.

### 2.3 Eligibility Criteria

#### 2.3.1 Study Selection

Titles and abstracts of all records identified through the database search were screened by two reviewers against the eligibility criteria. Full texts of potentially relevant articles were then independently assessed in duplicate. Discrepancies were resolved through discussion, and if consensus was not achieved, a third reviewer adjudicated. Screening was conducted in accordance with PRISMA 2020 guidelines.

#### 2.3.2 Inclusion Criteria

Eligibility criteria stipulated that the following requirements were satisfied for inclusion. Studies were required to be: (a) a randomised controlled trial design, (b) describing the implementation of a psychological intervention for BPD, (c) include children and/or adolescents (0–18) or young adults (18–25), (d) include participants who were experiencing BPD symptoms, and reported any outcomes of functioning (e.g., including social, occupational and vocational) as defined by the author(s). Consequently, functional outcomes were not predefined and were instead determined by the author(s).

#### 2.3.3 Exclusion Criteria

Due to resource constraints, the review focused solely on English‐language papers published between 1980 (the year when BPD was first described in the DSM‐III by APA) (American Psychiatric Association 1987) and the search date of 30th June 2023, ensuring a specific time frame for the included studies. Exclusion criteria were applied to studies that did not involve the use of a psychological intervention. For the purposes of this review, a psychological intervention was broadly defined as a structured and targeted therapeutic process that encompasses communication between one or more individuals and a trained practitioner.

### 2.4 Assessment of Quality

To evaluate the methodological strength and clinical applicability of the studies examined, the Cochrane Risk of Bias Tool 2 for Randomised Controlled Studies (Higgins et al. 2016) was used for this review. See Table 1.

**TABLE 1:** Overview of measures of functioning.

Study	Measure	Administration	Functional domains assessed	Scoring
Chanen et al. (2008) Gleeson et al. (2012)	SOFAS	Clinician or observer‐rated based on knowledge of patient or interview	Social (interpersonal) and occupational performance	On the SOFAS, the individual is provided with a score out of 100 which considers social, occupational and/or academic functioning. A higher score indicates a higher level of functioning
Pistorello et al. (2012), Asarnow et al. (2021), Chanen, Betts, et al. (2022)	SAS‐SR	Self‐reported structured questionnaire with 5‐point Likert scale	Social (relationships with family and extended family and leisure activities) emotional adjustment and school or work	SAS‐SR contains 54 items that assess role performance over the past 2 weeks. Six domains reviewed including work/school, social/leisure activities, extended family, primary relationship, parental role and family unit
Chanen, Sharp, et al. (2022)	IIP‐C	Self‐reported structured questionnaire with 5‐point Likert scale	Social (interpersonal)	IIP is a 64‐item measure designed to assess interpersonal difficulties. Items organised in a circumplex structure. The dimensions include dominance, submission, hostility, warmth, aloofness, nurturance, manipulation and social avoidance
Mehlum et al. (2016) Jørgensen, Storebø, Bo, et al. (2021)	CGAS	Clinician or observer‐rated based on knowledge of patient or interview	Social (interpersonal) and academic performance	The CGAS is scored on a scale ranging from 1 to 100, with higher scores indicating better overall functioning. The CGAS considers numerous domains, including academic performance, interactions with family and peers, emotional well‐being

Abbreviations: CGAS, Children's Global Assessment Scale; IIP‐C, Inventory of Interpersonal Problems—Circumplex version; SAS‐SR, Social‐Adjustment Scale—Self‐Report; SOFAS, Social and Occupational Functioning Assessment Scale.

### 2.5 Data Extraction

A data extraction proforma was created and piloted. Study details (authors, year, title, journal, country, sample size) and demographic data (age, gender, ethnicity, diagnosis, setting) were extracted. The primary outcome was the impact of psychological intervention on functioning for children and young adults with BPD symptoms. Functioning was broadly defined as described by study authors, encompassing social, occupational, leisure and global functioning measures. Intervention characteristics, treatment effects and functioning measures were tabulated and summarised in Table 1.

### 2.6 Statistical Analyses

A primary aim was to present a meta‐analysis of the overall effect of psychological intervention on functioning in adolescents and young adults with BPD features. We summarise the effect of intervention on functioning by examining the treatment effect at post‐treatment and final follow‐up. To ensure comparability of different outcome measures, standardised mean differences (SMD) were computed in the form of Hedge's *g* using the approach described by Hedges and Olkin (1985).

The meta‐analysis was performed using the R software and the Metafor package (Viechtbauer 2010), with a random‐effects model using restricted maximum likelihood estimation to measure between‐study variance and producing a Wald‐type confidence interval. Heterogeneity among studies was assessed using the *I* ^2^ statistic to quantify the proportion of total variation across studies due to heterogeneity rather than chance.

SMDs were calculated from pre‐ to post‐intervention and follow‐up change scores to compare the experimental and matched TAU groups. The meta‐analysis aimed to assess differences in functioning at post‐intervention and final follow‐up. Pooled SMDs were interpreted as small (0.2), medium (0.5) and large (0.8) effects (Hedges and Olkin 1985).

## 3 Results

**FIGURE 1:**
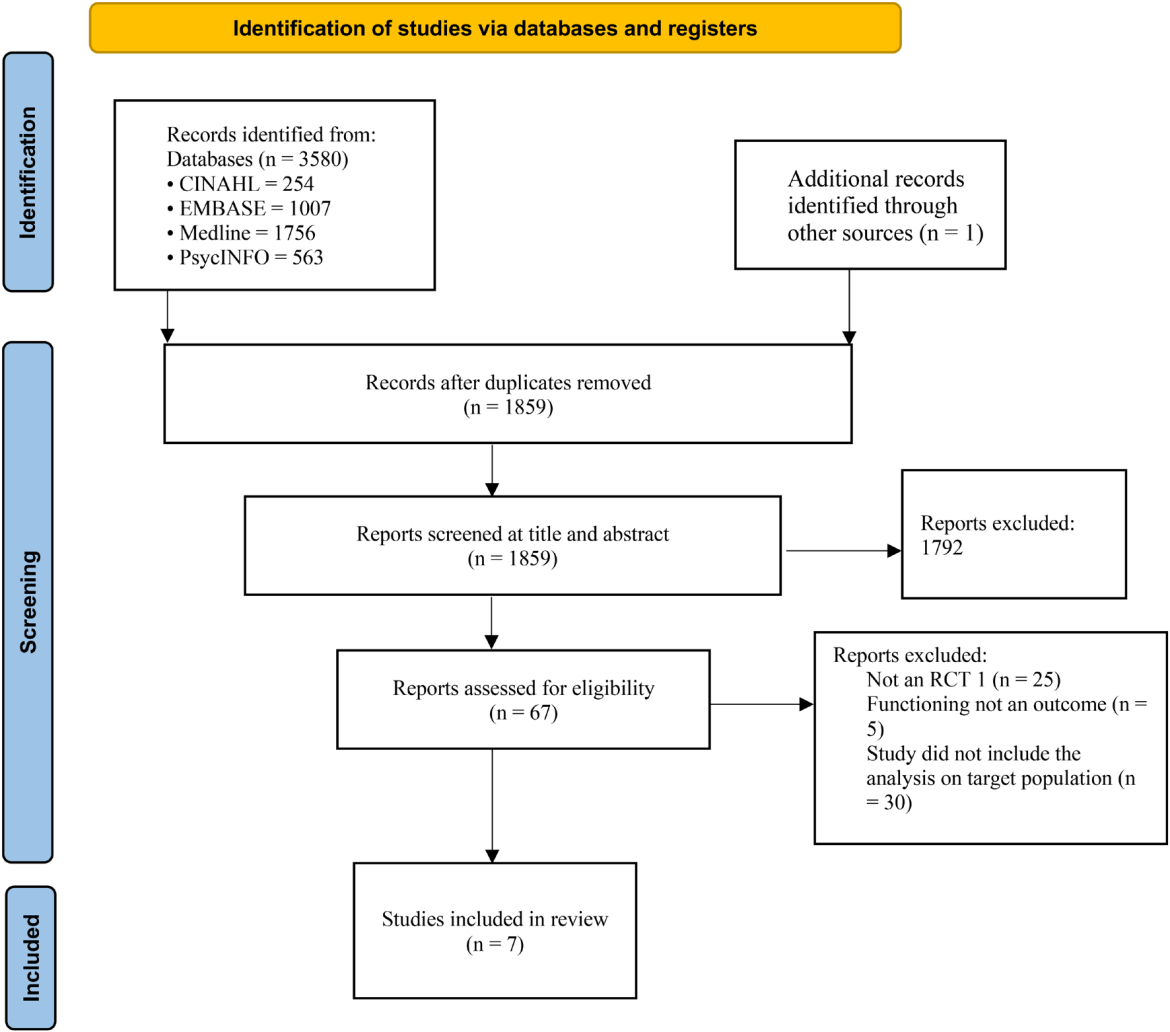
PRISMA flow diagram illustrating the identification, screening, eligibility assessment and final inclusion of studies for the review.

The search strategy yielded 3580 citations. After removing 1722 duplicates and adding one article through hand searching, 1859 records were screened at the title and abstract level. Articles were excluded if they did not examine BPD, were not randomised controlled trials or did not test psychological interventions. Of 67 full‐text studies reviewed for eligibility, articles were excluded if they did not include children, adolescents or young adults with BPD symptoms or lacked functioning measures. Reference scanning of the final seven articles yielded no additional studies. See Figure 1 for the PRISMA flow diagram (Page et al. 2021). Table 2 provides details on the seven eligible articles included in this systematic review.

**TABLE 2:** Study characteristics.

Study	Trial design and setting	BPD criteria met (diagnostic framework)	Total n (%f/m)	Age, years (range and mean, SD)	Intervention	Comparison	Duration	Functional outcome (primary or secondary)	Time points	Effect on functioning and between group effect sizes at post‐treatment
Chanen et al. (2008)	Outpatient, Australia, RCT	> 2 and additional risk factor (DSM–IV)	78 76/24%	15–18 (16.4, 0.9)	CAT	GCC	24 weeks	SOFAS (primary)	0, 6, 12 and 24 months	Both groups improved from baseline in functioning, which was sustained at 24 months. No significant difference between groups at follow‐up until 24 months at which point GCC was better. Rate of change was quicker in CAT
Gleeson et al. (2012)	Outpatient, Australia, RCT	> 4 (DSM–IV)	16 81.25/18.75%	15–25 (18.4, 2.9)	CAT + SFET	SFET	17 weeks + 2 booster sessions	SOFAS (secondary)	0, EOT and 6 months	Significant improvement in functioning from baseline to EOT and 6 months. Experimental group had better functioning at 6 months and EOT
Pistorello et al. (2012)	Outpatient, USA, RCT	> 3 and least one act of lifetime NSSI and or suicide attempt (DSM–IV)	63 70.95/19.05%	18–25 (20.86, 1.92)	DBT	O‐TAU	12 months	SAS‐SR (secondary)	0, 3, 6, 9, 12 and 18 months	Significant improvement between baseline and all timepoints on both conditions (symptoms and functioning). Better improvement for experimental condition compared to those in the comparison condition at post‐treatment and final follow‐up
Mehlum et al. (2016)	Outpatient, Norway, RCT	> 2 and history of at least two episodes of self‐harm, at least one episode within the last 16 weeks (DSM–IV)	77 88.31/11.69%	12–18 (15.6, 1.5)	DBT‐A	EUC	19 weeks	C‐GAS (secondary)	0, 19 and 71 weeks	Both groups showed significant improvement in functioning at post‐treatment and at 71 weeks. Minimal difference between experimental and control group in functioning
Asarnow et al. (2021)	Outpatient, USA, RCT	> 3 and at least 1 lifetime suicide attempt, elevated past‐month suicidal ideation (DSM–IV)	173 94.22/5.78%	12–18 (14.89, 1.47)	DBT	IGST	6 months	SAS‐SR (secondary)	0, 3, 6, 9 and 12 months	Both groups showed significant improvement post‐treatment and at 12 months. DBT group showed better functioning but were less severe at baseline
Jørgensen, Storebø, Bo, et al. (2021)	Outpatient, Denmark, RCT	> 4 (DSM–5) + > 67 on BPFS‐C	111 99.1/0.9%	14–17 (15.8, 1.1)	MBT‐G	TAU	12 months	C‐GAS (secondary)	0, 3 times during treatment phase, EOT, 3 and 12 months post‐treatment	Both groups showed improved function between baseline and 12 months. No difference found between experimental condition and TAU on functioning. At end of trial both groups were rated as having ‘variable functioning with sporadic difficulties or symptoms
Chanen, Betts, et al. (2022)	Outpatient, Australia, RCT	> 5 (DSM‐IV‐TR)	139 80.58/19.42%	15–25 (19.1, 2.8)	HYPE + CAT	HYPE + BEF; YMHS + BEF	16 sessions (16–25 weeks)	IIP; SAS‐SR (primary)	0, 3, 6, 12 and 18 months	All groups improved significantly on both measures of functioning and 12 months. These benefits were sustained with the comparison group (YMHS + BEF) outperforming the active therapy conditions on the IIP‐C, but not the SAS at the end of the trial follow‐up

Abbreviations: BEF, befriending; BPFS, Borderline Personality Features Scale for Children; CAT, cognitive analytic therapy; C‐GAS, Children's Global Assessment Scale; DBT, dialectical Behaviour therapy; DBT‐A, dialectical behaviour therapy for adolescents; DSM, Diagnostic and Statistical Manual of Mental Disorders; EOT, end of treatment; GCC, general clinical care; HYPE, Helping Young People Early; IGST, Intensive Group Skills Training; IIP, inventory of interpersonal problems; MBT‐G, mentalisation‐based treatment—group format; O‐TAU, optimised treatment as usual; SAS‐SR, Social Adjustment Scale—Self‐Report; SFET, specialist first episode treatment; SOFAS, Social and Occupational Functioning Assessment Scale; TAU, treatment as usual; USA, United States of America; YMHS, Youth Mental Health Service.

### 3.1 Study Characteristics

Seven RCTs were identified from the search. Studies were undertaken in: Australia (*n* = 3), USA (*n* = 2), Norway (*n* = 1) and Denmark (*n* = 1). Eligible studies reported data for 657 participants (86.71% female). The mean age ranged from 14.89 to 20.86. All samples were composed of adolescents or young adult outpatients receiving care in the community. Only three studies reported data on ethnicity. Of these, Pistorello et al. (2012) reported ethnicity for the full sample (69.8% ‘White’; 6.3% ‘Asian American’; 11.1% Hispanic; 31.7% ‘African American’ and 4.8% ‘Native American’) as well as Asarnow et al. (2021) (56.39% ‘White’; 5.85% ‘Asian American’; 27.49% Hispanic; 7.02% ‘African American’ and 0.58% ‘Native American’). Mehlum et al. (2016) reported that 84.9% of their sample was of ‘Norwegian ethnicity’. Two studies identified functional outcomes as primary outcomes (Chanen, Betts, et al. 2022; Gleeson et al. 2012).

### 3.2 Criteria for Assessing BPD Features and Inclusion

All studies in the sample refer to DSM criteria, with the DSM‐IV being the most common (American Psychiatric Association 1994). However, the thresholds at which participants were accepted into the studies varied significantly. Four studies required that participants met at least subthreshold criteria, thus having three symptoms present or more (Chanen, Betts, et al. 2022; Gleeson et al. 2012; Jørgensen, Storebø, Bo, et al. 2021; Pistorello et al. 2012). Three studies required an additional risk factor such as self‐harm behaviour, low socio‐economic status or history of abuse/neglect (Chanen et al. 2008; Mehlum et al. 2016; Asarnow et al. 2021). Chanen et al. (2008) specified additional risk criteria such as low socio‐economic status or experience of previous abuse or neglect. Whereas Mehlum et al. (2016) required at least three BPD features as well as one episode of self‐harming behaviour two weeks prior to entry. Similarly, Asarnow et al. (2021) required three or more BPD features, at least one suicide attempt, three or more episodes of self‐harm over the individual's life and ≥ 24 on the Suicidal Ideation Questionnaire‐Junior.

### 3.3 Measures of Functioning and Method of Assessment

Refer to Table 1 for specific measures of functioning and administration details. Four functioning measures were identified across the seven studies: the SOFAS, the C‐GAS, the SAS‐SR, and the IIP‐C. Functioning was a primary outcome in two studies (Chanen et al. 2008; Chanen, Betts, et al. 2022), with one study (Chanen, Betts, et al. 2022) examining multiple domains using two measures (SAS‐SR; IIP‐C). Two studies utilised the SOFAS (Chanen et al. 2008; Gleeson et al. 2012), while two employed the C‐GAS (Jørgensen, Storebø, Bo, et al. 2021; Mehlum et al. 2016). All studies assessed social functioning and either educational or vocational functioning, though assessment approaches varied. Three studies used structured questionnaires prompting participant feedback on skills and activity frequency (Pistorello et al. 2012; Asarnow et al. 2021; Chanen, Betts, et al. 2022), while four employed single‐score scaled summaries through semi‐structured interviews (Chanen et al. 2008; Gleeson et al. 2012; Jørgensen, Storebø, Stoffers‐Winterling, et al. 2021; Mehlum et al. 2016).

### 3.4 Intervention

A diverse range of therapies was observed among selected studies. Three studies (Chanen et al. 2008; Gleeson et al. 2012; Chanen, Betts, et al. 2022) utilised Cognitive Analytic Therapy (CAT) as the primary intervention, which was integrated with the Helping Young People Early (HYPE) model. The HYPE programme is a specialised treatment programme that adopts a multidisciplinary approach, incorporating relational clinical care, case management, general psychiatric care and talking therapy (Chanen et al. 2008). Three studies (Mehlum et al. 2016; Asarnow et al. 2021; Pistorello et al. 2012) used DBT‐A or DBT and employed similar intervention methods, including weekly individual therapy sessions, multifamily skills training, and telephone coaching with therapists outside of therapy sessions. Jørgensen, Storebø, Bo, et al. (2021) implemented mentalisation‐based treatment (MBT) in a group format, comprising three introductory sessions, five individual case formulation sessions, 37 weekly group sessions and six sessions with parents.

There were variations in the duration of the interventions delivered across selected studies. Mehlum et al. (2016) conducted their intervention over 19 weeks, whilst Asarnow et al. (2021) delivered intervention over a 6‐month period. Two other studies, Pistorello et al. (2012) and Jørgensen, Storebø, Bo, et al. (2021), completed their treatments over 1 year. Whereas Chanen et al. (2008, 2022) and Gleeson et al. (2012) did not specify explicit time frames for their interventions; instead, they indicated the number of sessions, which were 16, 13 and 17 sessions, respectively.

### 3.5 Comparison Condition

All papers included an active treatment condition as a control, with variations in the control condition therapy. Studies compared it to different descriptions of TAU or good clinical care (Chanen et al. 2008; Jørgensen, Storebø, Bo, et al. 2021; Mehlum et al. 2016). Descriptions of TAU or good clinical care varied between studies. Two studies discussed non‐specific conditions integrating either psychodynamic or cognitive‐behavioural strategies (Pistorello et al. 2012; Mehlum et al. 2016).

Jørgensen, Storebø, Bo, et al. (2021) described TAU as a non‐manualised approach that included psychoeducation, counselling, crisis management and caregiver participation. However, sessions were conducted monthly. Chanen, Sharp, et al. (2022) compared the active treatment condition to two interventions within the HYPE model, one using befriending and the other integrating HYPE with a Young Mental Health Service (YMHS). Asarnow et al. (2021) compared the active treatment condition to a general “individual and group supportive therapy” focused on addressing “thwarted belongingness,” emphasising acceptance, validation and fostering a sense of connection and belonging, with ad hoc sessions involving parents.

### 3.6 Quality Appraisal

Quality appraisals using the ROB2 of included studies are detailed in Table 3. Thus, studies were assessed for randomisation process, deviations from intended interventions, missing outcome data, measurement of outcomes and selection of reported results. Reviewers followed the guidelines provided by the Cochrane Collaboration to assign judgements of low, some concerns or high risk of bias for each domain. Study quality was rated low (*n* = 2), some concerns (*n* = 4) and high (*n* = 1).

**TABLE 3:** Risk of bias tool 2 (ROB2).

	Study	Domain 1	Domain 2	Domain 3	Domain 4	Domain 5	Overall RISK OF BIAS
		Randomisation process	Deviations from intended interventions	Missing outcome data	Measurement of the outcome	Selection of the reported result	
1	Chanen et al. (2008)	Low	Low	Some concerns	Low	Some concerns	Some concerns
2	Gleeson et al. (2012)	Low	Some concerns	High	Some concerns	Some concerns	High
3	Pistorello et al. (2012)	Some concerns	Low	Low	Low	Some concerns	Some concerns
4	Mehlum et al. (2016)	Some concerns	Low	Low	Low	Some concerns	Some concerns
5	Asarnow et al. (2021)	Low	Low	Low	Low	Low	Low
6	Jørgensen, Storebø, Bo, et al. (2021)	Low	Low	Some concerns	Low	Low	Some concerns
7	Chanen, Sharp, et al. (2022)	Low	Low	Low	Low	Low	Low

*Note:* Green highlight indicate the Low risk of bias, Yellow/Amber highlight indicate Some concerns, and Red highlight indicate the High risk of bias.

To ensure consistent quality appraisal and establish inter‐rater reliability, authors independently assessed all seven papers. Initially, there was a weighted κ agreement of 0.818, which indicated substantial agreement. The use of a weighted kappa score was appropriate as the evaluated categories had an inherent order or hierarchy. Although there was initially a discrepancy one paper, following discussion, complete agreement was reached.

### 3.7 Meta‐Analysis of Functional Outcomes

Chanen, Sharp, et al. (2022) included three conditions, where the experimental condition CAT + HYPE was compared with an active TAU‐like condition, HYPE + YMHS. Two measures of functioning were used: the IIP‐C (interpersonal functioning) and the SAS‐SR (social adjustment, leisure, educational and vocational). For consistency and broader scope, the SAS‐SR was selected as the primary outcome measure, as it was also used in three other studies, enhancing comparability and validity.

#### 3.7.1 Effect of Psychological Interventions on Functional Outcomes at Post‐Treatment

**FIGURE 2:**
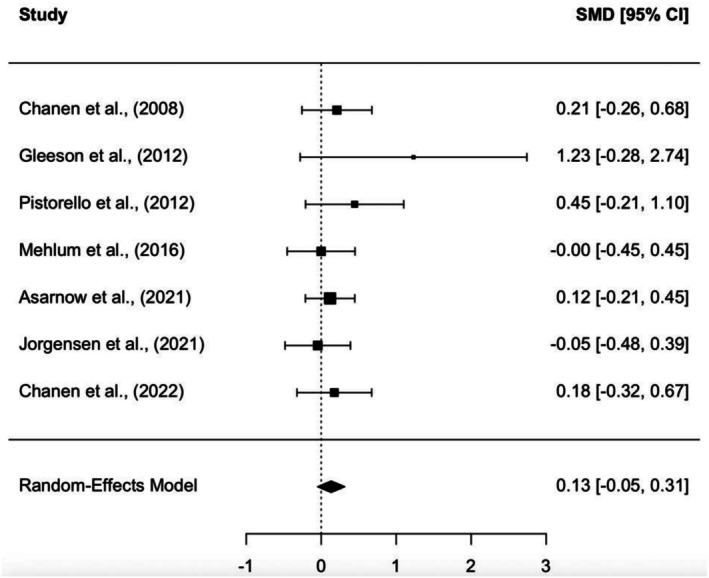
Forest plot of post‐treatment effect sizes.

Seven studies (*N* = 506) were included in a meta‐analysis of post‐treatment effect sizes (SMD). Specialised psychological interventions did not significantly improve functional outcomes compared to control groups, *p* = 0.148. The meta‐analysis yielded a small effect size favouring the intervention group (SMD = 0.13, 95% CI [−0.05, 0.31]). Effect sizes for individual studies ranged from −0.30 to 1.38, with negligible heterogeneity observed (*T* ^2^ < 0.001, *Q*[6] = 4.03, *p* = 0.673, *I* ^2^ < 1%; see Figure 2 for the forest plot). Excluding the high‐risk study in a sensitivity analysis did not substantially change the pooled effect size (SMD = 0.12, 95% CI [−0.06, 0.30]) or heterogeneity.

#### 3.7.2 Effect of Psychological Interventions on Functional Outcomes at Final Follow‐Up

**FIGURE 3:**
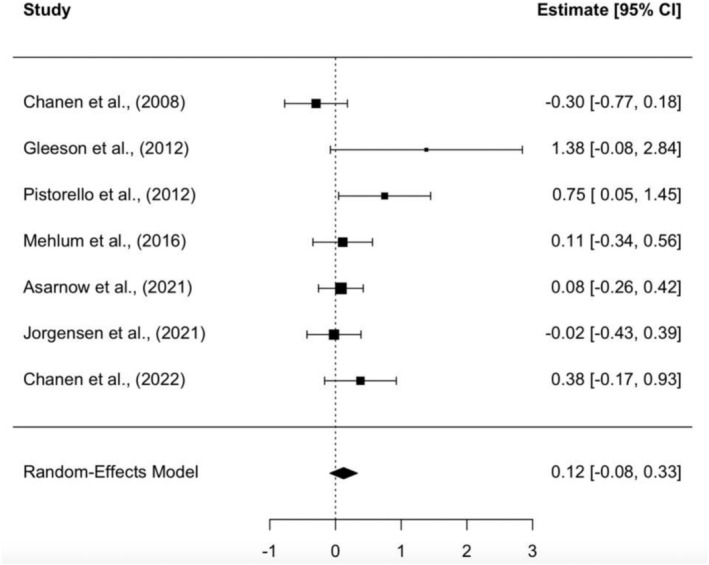
Forest plots for effect size at final follow‐up.

Seven studies (*N* = 508) were included in a meta‐analysis of final follow‐up effect sizes (SMD). Specialised psychological interventions again did not significantly improve functional outcomes compared to control groups, *p* = 0.245. The overall effect size at follow‐up was SMD = 0.12, 95% CI [−0.08, 0.33], consistent with post‐treatment findings. Effect sizes for individual studies ranged from −0.30 to 1.38, with low heterogeneity observed (*T* ^2^ = 0.014, *Q*[6] = 10.24, *p* = 0.115, *I* ^2^ = 17.5%; see Figure 3 for the forest plot). Excluding the high‐risk study in a sensitivity analysis had minimal impact on the pooled effect size (SMD = 0.09, 95% CI [−0.10, 0.27]) or heterogeneity.

#### 3.7.3 Overall Effect on Functioning

All studies showed that participants improved in functioning from baseline to the end of the trial. However, only two studies (Gleeson et al. 2012; Pistorello et al. 2012) found a significant positive effect of the experimental condition on functioning compared to the control condition. Both studies were rated as having some concerns or a high risk of bias. Only two studies were identified as having a low risk of bias.

At post‐treatment, Chanen et al. (2008) reported that the experimental group had a higher level of functioning (SMD = 0.26). However, at the 24‐month follow‐up, participants in the Good Clinical Care (GCC) condition exhibited higher overall functioning levels.

Post‐treatment effects on functioning were small overall and varied across studies. Pistorello et al. (2012) reported a medium effect (SMD = 0.45), Asarnow et al. (2021) reported a small effect (SMD = 0.12), and Mehlum et al. (2016) reported no effect (SMD = 0.00). At final follow‐up, effects remained small in Mehlum et al. (2016; SMD = 0.11) and Asarnow et al. (2021; SMD = 0.08), whereas Pistorello et al. (2012) showed a medium‐to‐large effect (SMD = 0.75) (Table 3).

Jørgensen, Storebø, Bo, et al. (2021) reported very small effect sizes at both time points. At post‐treatment, the effect slightly favored the control TAU condition (SMD = −0.05), and at final follow‐up, the effect was near zero (SMD = −0.02).

Chanen, Betts, et al. (2022) reported improvements in functioning across all three conditions at both post‐treatment and final follow‐up. While all conditions showed gains on both the IIP‐C and SAS‐SR, planned comparisons revealed no significant differences between service models (YMHS + befriending vs. HYPE) or psychotherapy interventions (HYPE + CAT vs. befriending) at 12 months, regardless of the functional outcome measure used. Functional improvements continued through to final follow‐up across all conditions.

## 4 Discussion

This systematic review and meta‐analysis are the first comprehensive examination of the effectiveness of psychological interventions in improving functioning among adolescents and young adults with BPD features. We aimed to systematically analyse and synthesise existing literature on targeted psychological interventions and their impact on functioning and assess the quality of the evidence in this population.

### 4.1 Effectiveness of Interventions on Functioning

The main findings suggest that while functioning improved across all groups, specialised psychological interventions did not lead to consistently greater improvements when compared with control conditions. In some studies, control conditions such as TAU showed comparable or even slightly better outcomes at post‐treatment. For example, Chanen et al. (2008) found that the ‘Good Clinical Care’ condition outperformed the experimental CAT intervention at the 24‐month endpoint. In this meta‐analysis, no single intervention type emerged as clearly superior across studies. However, the quality of evidence as measured by the risk of bias was variable. This is with the exception of two studies which were rated as ‘low’. While there was an observed improvement in functioning, it falls outside the scope of this review to ascertain whether functioning reached levels to be expected at the respective developmental phase.

Given the high cost and variable outcomes of specialised psychological interventions, generalist models may offer a more pragmatic and scalable solution. Leichsenring et al. (2011) highlighted the high societal costs associated with BPD and the lack of clear superiority among specific psychotherapies. In line with this, Bateman and Krawitz (2013) have argued that generalist approaches such as Structured Clinical Management (SCM) can be delivered effectively by general mental health professionals and may yield comparable outcomes to specialist treatments.

Interventions that directly target role and social functioning may be particularly valuable, potentially offering more efficient gains in functional domains than psychotherapy alone. However, the quality of evidence was mixed, with only a minority of studies rated as low risk of bias. While functioning improved overall, this review did not assess whether participants reached developmentally normative levels of functioning.

The ongoing INVEST Trial is evaluating the effects of individualised placement and support on functioning in young people with BPD features, through personalised assistance and sustained educational and vocational support (Chanen, Nicol, Betts, Bond, et al. 2020). Integrating the Individual Placement and Support model with psychological intervention, the trial emphasises rapid engagement in meaningful roles and time‐limited support. By prioritising functioning as a primary outcome rather than a secondary one, INVEST embodies a pragmatic ‘functional‐first’ approach consistent with early intervention principles in BPD.

Chanen, Betts, et al. (2022) posited that psychotherapy might not be the most suitable early‐stage treatment for BPD, and instead, it might be more appropriate for individuals with nonacute or BPD features, in comparison to later stages of the disorder or individuals with more developed self‐regulatory capacities. This is in line with the clinical staging model (McGorry et al. 2006; Minnis et al. 2022), whereby intervention should match the symptom severity, functional impairment and duration. Recently, Hutsebaut et al. (2020) considered a staging model for BPD, which highlights the presence of functional difficulties from the early stages and persisting into the late stages.

Contrary to a staged model or linear progression, personalised interventions targeting specific features of BPD may result in enhanced functional outcomes, as certain features have been shown to predict functional impairment. For example, Juurlink et al. (2022) found that identity instability and chronic emptiness predicted vocational difficulties, while perceived social support has been shown to act as a protective factor against functional impairment (Thadani et al. 2022). As such, it is important to be open to all forms of trials and interventions for BPD in adolescents and young adults as the work is still in its nascent stages in clinical and community settings (Gajwani et al. 2022). The awaited ODDESSI trial, an RCT exploring Open Dialogue Therapy's effects, a social network model of crisis and continuing mental healthcare, could offer valuable insights into the impact on functioning and recovery (Pilling et al. 2022).

Findings indicated that effect sizes remained stable from post‐treatment to final follow‐up for longer‐term outcomes in specialist BPD interventions. However, the varying time intervals between post‐treatment and follow‐up suggest a need for cautious interpretation. If both the standard and targeted interventions lead to similar improvements in functioning, it may suggest that preserving functional gains over time depends more on factors such as support and follow‐up care rather than the specific type of intervention used.

Importantly in this review, most studies considered functioning as a secondary outcome, and the five studies that met all other criteria were excluded for not assessing functioning. This is an important finding, as it indicates that functioning is still not prioritised as a key outcome despite the literature base demonstrating that functional difficulties are widespread in this population (Videler et al. 2019).

### 4.2 Outcome Measures of Functioning

A mixture of self‐report and clinician‐rated outcomes were used across all seven studies. Although all studies used standardised and well‐validated measures, there were limitations to the methods of assessment.

The C‐GAS, like the Global Assessment of Functioning (GAF), integrates symptom‐based outcomes into its overall score. However, functional impairment has been reported even with good symptom‐based outcomes (Biskin et al. 2011; Gunderson et al. 2011), highlighting the importance of separating these domains. This led to the development of the SOFAS, which focuses exclusively on social and occupational functioning without symptom conflation. Yet, given the breadth of BPD features, identifying comprehensive assessments unbiased by symptomatology remains challenging, as functioning is conceptually intertwined with symptoms. Furthermore, debate persists about whether functioning can be reliably assessed by clinicians, given that social inclusion and recovery may be more appropriately measured by individuals themselves due to their subjective and experiential nature (Burgess et al. 2017).

These conceptual debates are compounded by practical measurement challenges. Relying on a single score to assess overall functioning can introduce biases and limitations, as functioning is multifaceted and spans diverse domains such as social relationships, academic performance, quality of life, and capabilities. The World Health Organisation's International Classification of Functioning prioritises functioning over disability, emphasising 'activity' (task performance) and 'participation' (engagement in life roles), reflecting a comprehensive approach to capturing its complexity (World Health Organization 2001). Gerber and Price (2018) conceptualise functional status as the degree to which an individual can perform chosen roles without limitation across three key domains: physical, social, and psychocognitive. For adolescents and young adults with BPD, functional measures should be carefully matched to developmental stage, ensuring assessments are relevant and sensitive to the unique capabilities and challenges characteristic of this period. This developmental sensitivity is essential to accurately capture functioning in this population.

### 4.3 Strengths and Limitations

This systematic review has followed current best practices through registration with PROSPERO and has followed standard reporting procedures as per PRISMA guidelines. Moreover, this review provides a summarisation of an often marginalised and underrepresented group across a broad early intervention‐based age range.

Several factors limited the generalisability of the evidence. Notably, participant ethnicity was poorly reported across the included studies. In addition, the quantitative focus of the review precluded consideration of participants’ subjective experiences of intervention. Furthermore, the predominance of female samples limited the extent to which findings can be generalised.

Furthermore, three out of the seven studies primarily focused on self‐harming behaviour or suicidality, potentially introducing sampling bias and restricting the applicability of the findings. Although non‐suicidal self‐injury and suicidal behaviour are common features of BPD, indeed, subthreshold and threshold adolescent BPD occurs in the absence of this feature. However, often suicidal expression is the strongest impetus for treatment (Zimmerman and Becker 2022). Additionally, variations in BPD phenotype have been found to exist between those that have suicidal ideation and behaviour as a feature compared to those that do not (Chabrol et al. 2004; Becker et al. 2006).

The current synthesis focused exclusively on randomised trial papers published in academic journals, which may have introduced publication bias, as unpublished studies or grey literature reporting non‐significant findings were not included. Despite a comprehensive search strategy and broad inclusion criteria, it is possible that ongoing or unpublished studies were not captured in this analysis. Furthermore, a substantial body of evidence examining longitudinal outcomes of functioning was not included in this review. As this study was restricted to randomised controlled trials, naturalistic and nonrandomised studies were excluded; however, such data are critical for understanding how interventions function in real‐world settings. Finally, the reliance on English‐language studies may have introduced a bias towards Western countries, potentially limiting the applicability and breadth of the findings.

The meta‐analysis assessed the impact of specialised psychological interventions on functional outcomes in adolescent BPD. Due to the limited number of studies, conducting comprehensive subgroup and meta‐regression analyses to address observed heterogeneity was not feasible. However, the similar outcomes observed across different intervention types (DBT vs. CAT) align with Common Factors Theory (Lambert et al. 1992), suggesting that shared therapeutic elements may contribute to efficacy across modalities.

A further limitation could be understood in the conceptualisation of functioning. Functioning was assessed in a broad, global sense, which may obscure differences between specific domains such as vocational, social and academic functioning. This broad operationalisation could limit the ability to identify whether certain interventions may preferentially improve one domain of functioning over another.

Finally, high heterogeneity between studies reflects substantial variation in study designs, interventions, outcome measures and follow‐up timepoints, which may limit the meaningfulness of pooled effect estimates. This variability is particularly problematic for long‐term follow‐up assessments given the rapid developmental changes characteristic of adolescence. Interpretations should therefore consider this heterogeneity when evaluating findings.

## 5 Conclusions

Creating scalable methods to address the short and long‐term adverse outcomes faced by young people with BPD is a public health priority (Holmes et al. 2020), with functioning emerging as a key treatment target among this group (Winsper 2021). This review found that although both experimental and control groups demonstrated improvements in functioning, specialised psychological interventions did not produce significantly greater benefits than TAU. Effect sizes tended to be within the small range, and it remains unclear what specific moderators were associated with functional improvements. The observed changes may be attributable to treatment effects or the natural progression of BPD features. Given the underrepresented and marginalised nature of this population, continued assessment of functional outcomes through high‐quality trials with larger sample sizes remains crucial for understanding effective interventions for young people with BPD features.

### 5.1 Implications for Theory, Clinical Practice and Future Research

While existing evidence demonstrates impaired functioning in BPD, the precise elements required for successful psychological interventions to enhance functioning remain uncertain. These findings highlight the need for service providers to consider this population's distinctive requirements and tailor interventions accordingly. Providers should recognise that functional difficulties persist throughout adolescence and adulthood and may benefit from generalist approaches. Further research should integrate young people's perspectives on crucial factors related to functioning and recovery, particularly given the notable dropout rates highlighted in this review. Research assessing psychological therapies in adolescent BPD should prioritise functional recovery as a primary outcome.

## Funding

Funding provided by NHS Greater Glasgow & Clyde and the University of Glasgow. Neither funding source had any involvement in the design, collection, analysis and interpretation of data or the decision to submit the article for publication.

## Data Availability

Data sharing not applicable to this article as no datasets were generated or analysed during the current study.
